# *In silico*, anti-inflammatory and acute toxicological evaluation of an indigenous medicinal plant *Pterospermum rubiginosum* using Sprague-Dawley rats

**DOI:** 10.1186/s42826-024-00191-w

**Published:** 2024-02-07

**Authors:** Rajamohanan Jalaja Anish, Aswathy Nair, V. Saraswathy, Velappan Nair S. Kalpana, Rajendran L. Shyma

**Affiliations:** 1 Department of Biochemistry, University of Kerala, Kariyavattom Campus, Thiruvananthapuram, Kerala 695581 India; 2 Kerala State Palmyrah Products Development and Workers’ Welfare Corporation Limited, Trivandrum, India; 3 Department of Computational Biology and Bioinformatics, University of Kerala, Trivandrum, Kerala India

**Keywords:** Anti-inflammatory, iNOS, LCMS, Molecular docking, *Pterospermum rubiginosum*, Sprague Dawley rats

## Abstract

**Background:**

*Pterospermum rubiginosum* has been traditionally used by the tribal inhabitants of Southern India for treating bone fractures and as a local anti-inflammatory agent; however, experimental evidence to support this traditional usage is lacking. The present study aimed to investigate the phytochemical characterization, *in silico* and in vitro anti-inflammatory evaluation, followed by in vivo toxicological screening of *P. rubiginosum* methanolic bark extract (PRME).

**Results:**

The LCMS evaluation revealed the presence of 80 significant peaks; nearly 50 molecules were identified using the LCMS database. *In silico* analysis showed notable interactions with inducible nitric oxide synthase (iNOS) and interleukin-6 (IL-6). In vitro gene expression study supported the docking results with significant down-regulation of iNOS, IL-6, and IL-10. PRME was administered orally to the SD rats and was found to be non-toxic up to 1000 mg/kg body weight for 14 days. The antioxidant enzymes catalase and sodium dismutase exhibited an increased value in PRME-administered groups, possibly due to the diverse phytochemical combinations in bark extract.

**Conclusions:**

PRME administration significantly downregulated the gene expression of inflammatory markers, such as iNOS, IL-6, and IL-10. The molecular docking analysis of iNOS and IL-6 supports the in vitro study. In vivo toxicological study of PRME in SD rats was found to be non-toxic up to a concentration of 1000 mg/kg body weight for 14 days.

**Graphical abstract:**

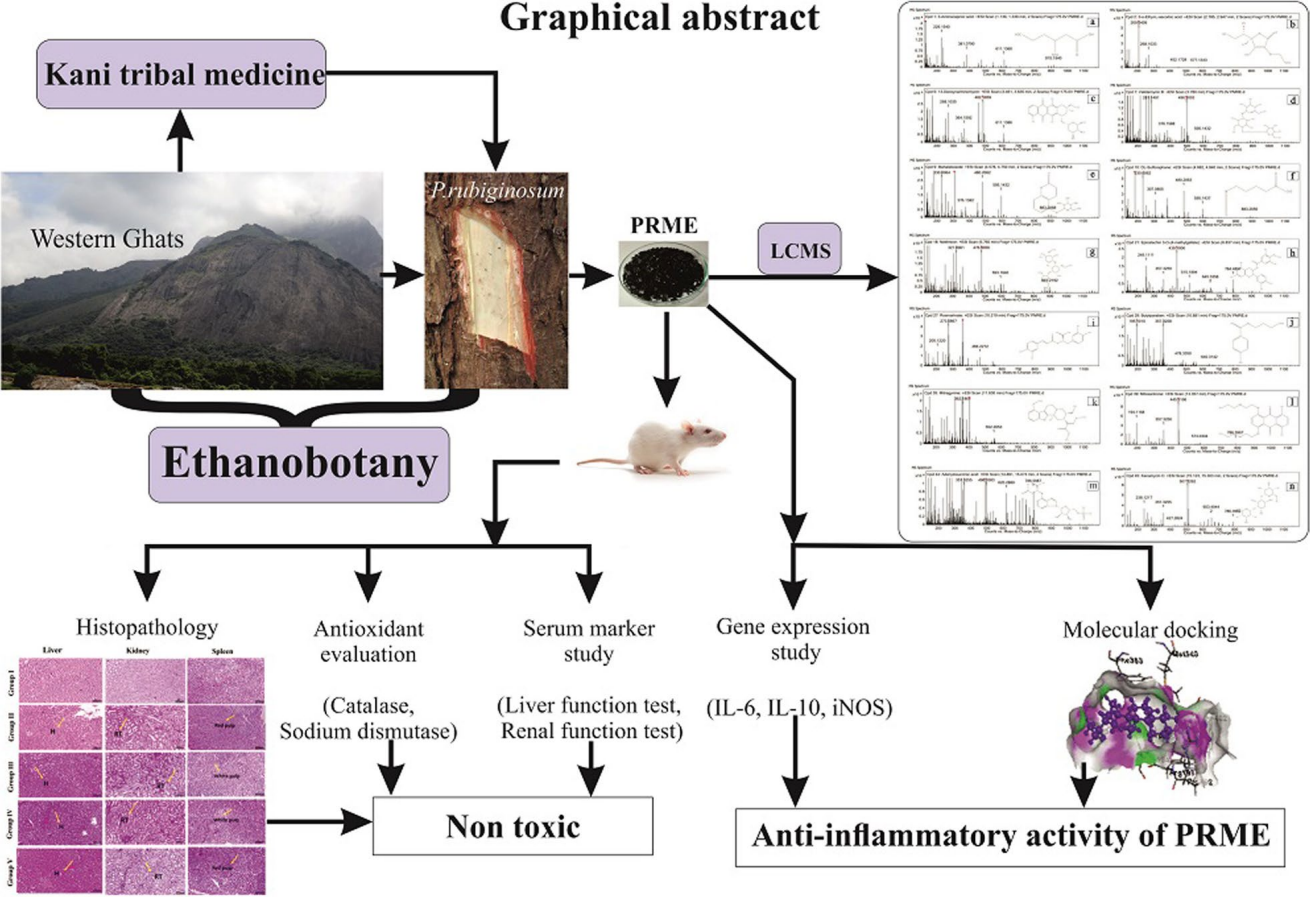

**Supplementary Information:**

The online version contains supplementary material available at 10.1186/s42826-024-00191-w.

## Background

Screening medicinal plants and natural product derivates for their toxicological properties is a fundamental criterion for developing new therapeutic agents and for evaluating the pharmaceutical efficacy and toxicity. Ordinary people believe that plant-based medicinal products are natural and, therefore, safe for consumption without any purification process. Without prior proper processing, the long-term usage of raw plant material, including crude extracts and herbal formulations, can cause severe toxicity, including organ damage [1]. Over the centuries, herbal formulations and phytomedicines have played a key role in improving the health of humankind in almost all well-known civilizations through their treatment systems, particularly Indian Ayurveda, ancient traditional Chinese medicine, and Greek Unani medicine [2].

Paracelsus states in the fundamentals of toxicology that any substance can be considered toxic if the consumption rate exceeds the permissible range [3]. According to WHO statistics, approximately two-thirds of the global population relies on herbal formulations or decoctions made from natural product derivatives to cure a variety of diseases. Even in affluent countries, people rely on plant-derived items for primary health care due to the severe contraindications and fatal effects of current synthetic pharmaceuticals. Unfortunately, the molecules extracted and described from these therapeutic formulations are not as effective as natural product combinations; this may be owing to the synergistic impact of several chemical groups of phytocompounds in plant extracts [4, 5].

Medicinal plant extracts have been used as traditional medicine by local residents of the Asian and African continents, particularly the people of India, China, and most African nations. Naidu et al. [6] investigated the long-term toxicity of medicinal plant products in indigenous communities. Most traditional wisdom is passed down through the generations as ancestral sayings [6]. Generally, plant extracts have not been thoroughly studied in terms of their safety. Modern medicine’s extensive development, as well as a lack of suitable guidance, experience, and information for traditional healers regarding the critical purification stage in raw natural product preparations, has hampered traditional medicinal practice in India. Secondly, most medicinal plants are becoming increasingly rare because of deforestation. In addition, soil pollution also increases the absorption of toxic compounds by plants, increasing the amount of toxins in medicinal plants. Due to the fact that these plants do not guarantee 100% safety for consumption, it is necessary to investigate and ensure the toxic effects of these plants on a long-term and short-term basis [7]. An international scientific committee was established by the World Health Organization [8] and the Food and Agriculture Organization of the United Nations (FAO) to assess the level of natural toxins in foods. Therefore, toxicity tests of natural products, including herbal formulations, must be conducted under in vitro and in vivo conditions, which are healthy practices to assess the toxic effects while enhancing the efficiency of herbal and natural products, which is vital to humanity.

Inflammation responses are considered an essential defence mechanism of the body against foreign bodies. These responses are crucial for the well-being of normal homeostasis. The molecular mechanism of inflammation is a multifactorial and complicated process. The pros and cons of inflammation in various pathways, including healing and pathological mechanisms, are already reported [9]. From this study, we planned to evaluate the anti-inflammatory ability of plant materials in RAW 264.7 cells. Any molecule that inhibits the inflammatory mediators without harming living beings can be considered a significant lead owing to the development of anti-inflammatory drugs. *In silico* screening and targeted docking studies will further evaluate these molecules to understand the druggability, cellular affinity, pharmacophores, and structure-activity interactions between various inflammatory proteins and ligand molecules [10].

Pterospermum is derived from two Greek words, Pteron, meaning “winged seed,” and Sperma, or “seed,” meaning “wing.” It is commonly known as Ellootti (in Malayalam) and Edinjal (in Tamil). Local Kani tribes use its bark as 'Ellooripatta' for its excellent bone regeneration potential. In the *Agasthya Vanam* region and Wayanad settlements, the stem bark of *P. rubiginosum* has been traditionally used to treat bone fractures and inflammation. A bark paste made from the inner bark of *P.rubiginosum* is applied to the fractured site with bamboo slides, similar to plaster or a bandage in modern medicine; the bark boiled in water is also offered to the patient to relieve inflammation at the fracture site. The leaf and bark extract mixed with warm oil for massage is commonly used in inflammation and pain relief treatment procedures [11, 12]. The toxicological evaluation of natural products can be assessed by acute, sub-acute, and chronic toxicity studies using experimental animals. Thus, the current study aimed to determine the *In silico* and in vitro anti-inflammatory analysis followed by quantifying the effective dose of *P. rubiginosum* bark in Sprague-Dawley rats. The PRME extract was administered according to OECD/OCDE guideline-423 at 50, 300, 500, and 1000 mg/kg/day doses for 14 days. After the PRME treatment, the animals were sacrificed to evaluate haematological and biochemical parameters.

## Methods

### Plant extraction

**Fig. 1:**
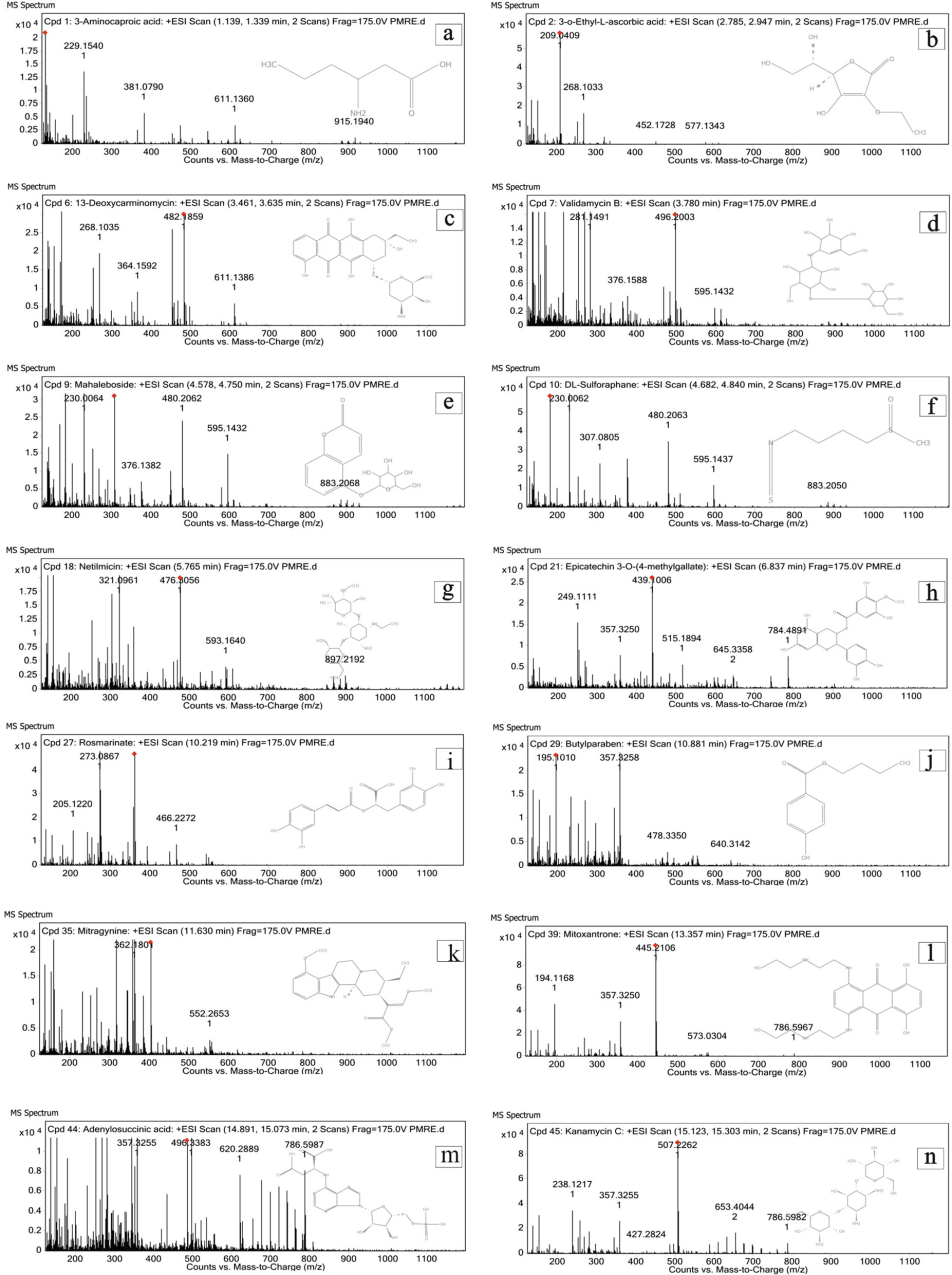
Biologically active compounds characterized from PRME using LCMS analysis; where **a** 3-Aminocaproic acid; **b** 3-O-Ethyl-L-ascorbic acid; **c** 13-Deoxycarminomycin; **d** Validamycin B; **e** Mahaleboside; **f** DL-Sulforaphane; **g** Netilmicin; **h** Epicatechin 3-O-(4-methylgallate); **i** Rosmarinate; **j** Butylparaben; **k** Mitragynine; **l** Mitoxantrone; **m** Adenylosuccinic acid; **n** Kanamycin C

*The pterospermum rubiginosum* (Malvaceae) family’s bark was collected from the Kottur forest range, Thiruvananthapuram district of Kerala (Western Ghats) with the help of tribal people. The curator identified the plant specimen and kept it in the herbarium of the Department of Botany, University of Kerala, Thiruvananthapuram, India, with a voucher number of KUBH 6189. After removing the exfoliated outer bark, the inner bark of *P. rubiginosum* was shade-dried for 3–4 weeks (Fig. 1). About 1000 g of the bark was powdered using a mixer grinder, and the fine powder material was passed through a 60-mesh sieve. The powder was extracted with methanol of high polarity using a Soxhlet apparatus, and the crude methanol extract was filtered using Whatman No. 1 filter paper, concentrated using a rotary evaporator, and the sample was kept at room temperature for further study [11].

### Liquid chromatography mass spectrometry (LCMS)

Q-Exactive Plus Biopharma-High Resolution Orbitrap liquid chromatography mass spectrometry (Thermo Fischer Scientific Pvt. Ltd.), equipped with a heated Electrospray Ionization (ESI) and Atmospheric Pressure Chemical Ionization (APCI), both soft ionisation techniques were used for investigating the detailed mass spectrum of phytoconstituents present in the PRME. The LCMS component model-G6550A, can achieve a scan speed of (30,000 µ/s) and a polarity switching speed (5 ms), which can attain ultra-high-speed, high-sensitivity analysis. 0.5 g of PRME was diluted with methanol and filtered with a 0.22 µm nylon filter, and 5 µl of the sample was injected into the analytical column. The LCMS unit was directly connected with Agilent Technologies version acquisition method info for the detailed analysis and mass fragmentation was identified by using a spectrum database for organic compounds.

### Molecular docking study

The three-dimensional structure of murine inducible nitric oxide synthase oxygenase (iNOS) and structure of mouse interleukins-6 (IL-6) were procured from PDB database with ID: 1DD7 and 2L3Y respectively and having a crystallographic resolution of 2.25 Å. The protein 1DD7 consists of one polypeptide chain (A) with 389 aminoacids. The protein (2L3Y) contains a polypeptide chains A, with 190 amino acids, having a molecular weight of 21.7 kDaltons. The active site of the protein interacting with the standardized ligand molecules was selected as the binding site, 9 poses in the case of 1DD7 and 6 poses (2L3Y) of the selected ligands in the docked complexes were generated for docking studies [13].

### Gene expression study

RAW 264.7 cell lines were seeded in 6 well plates at a density of 6–7 × 10^5^ cells/dish. After 24 h, the culture plate was changed with fresh medium, added PRME concentration of 50 µg/ml and leave untreated control cells, incubated for 24 h. Gene expression of iNOS, IL-6 and IL-10 were determined through a reverse transcription-polymerase chain reaction (RT-PCR) assay and GAPDH was used as a house keeping gene. Total ribonucleic acid (RNA) was extracted from samples with TRIzol reagent (Invitrogen, USA) [14]. The cDNA synthesis was performed using verso cDNA Synthesis kit. 4 µl of 5X cDNA synthesis buffer, 2 µl of dNTP, 1 µl of anchored oligodT, 1 µl of RT Enhancer, 1 µl of Verso Enzyme were mixed and 5 μl of RNA template (1 ng of total RNA) were added to an RNAse free tube, and the total volume made up to 20 μl using sterile distilled water. The thermal cycler was programmed to undergo cDNA synthesis. The following cycling conditions were employed, 30 min at 42 °C and 2 min at 95 °C. The reaction mixture of 50 µL consists of 25 µL of PCR master mix, 2 µL of forward and reverse primer, 5 µL of templates DNA and made up to 50 µL with sterile distilled water (nuclease-free). The denaturation step followed by annealing for 30 s and extension (72 °C for 1 min), repeated for 35 cycles and the final extension (72 °C for 5 min). After the amplification, the PCR product was separated by agarose gel electrophoresis. Oligonucleotide primer for PCR amplification of iNOS with forward (5′-CGAAACGCTTCACTTCCAA-3′) and reverse (5′-TGAGCCTATATTGCTGTGGCT-3′); IL-6 forward (5′-GATGCTACCAAACTGGATATAATC-3′) and reverse (5′-GGTCCTTAGCCACTCCTTCTGTG-3′); IL-10 forward (5′-CGGGAAGACAATAACTG-3′) and reverse (5′-CATTTCCGATAAGGCTTGG-3′) and GAPDH forward (5′-AGGGCTGCTTTTAACTCTGGT-3′); reverse (5′-CCCCACTTGATTTTGGAGGGA-3′) respectively. The primer sequences were procured from Biogene, New Delhi, India.

### Animal experiments

Healthy female Sprague-Dawley (SD) rats of 75–90 days age and 200–265 g body weight were procured from the animal house facility, Department of Biochemistry, University of Kerala. Before starting the study, the animals were acclimatized to the experimental conditions for 5–7 days, the rats were kept in clean cages under standard conditions such as temperature: 22 ± 3 °C; Relative Humidity: 50–60%; proper lighting with 12 h of light and dark cycle; animals were fed with standard laboratory diet and filtered water. Animals were treated as per CPCSEA guidelines, the experimental protocol was approved by the Institutional animal ethical committee (IAEC-2-KU-01/2018–19-BCH-AAR (13) and IAEC-KU-09/2018-19-BCH-AAR (12); the dose-dependent toxicity study was sanctioned to be conducted in agreement with OECD guidelines.

### Experimental groups

The experimental rats were grouped into 5 groups of six animals each and a single dose of 50, 300, 500, and 1000 mg/kg/day of PRME was orally administered to groups II, III, IV, and V, while group 1 control rats were provided (normal saline) for 14 days. On 15th day, the animals were sacrificed, blood was collected for biochemical analysis, and internal organs like the liver, spleen, and kidney were collected and preserved for further histopathological examinations.

### Biochemical and haematological parameters

The blood samples of experimental animals were collected in an EDTA tube for the evaluation of different blood parameters such as haemoglobin, red blood cell count (RBC count), white blood cell count (WBC count), packed cell volume (PCV) and platelet counts using standard methods. The in vitro quantitative determination of SGPT, SGOT, Total protein, albumin, serum urea, uric acid, cholesterol, and triglycerides were carried out using the diagnostic kits, (Agape Diagnostics, Kerala, India). SOD activity was determined by the method adapted from Kakkar et al. [15]. The catalase activity was measured by the protocol of Chance and Maehly [16]. The glutathione reductase, glutathione peroxidase, and glutathione content were assayed by the methods of David and Richard [17]. Lipid peroxidation was evaluated for determining the cellular oxidative stress levels and determined by measuring the secondary product of peroxidation, malondialdehyde (MDA), by the standard method of Ohkawa et al. [18], hydroperoxides by the iodometric method of Mair and Hall [18, 19]. Conjugated dienes were quantified by using the method of Recknagel and Ghoshal, with slight modifications [20].

### Histopathological examination of hepatic and renal tissues

Histopathological evaluations of the liver, spleen, and kidney were performed. The tissues were fixed in a 10% buffered neutral formalin solution and embedded in paraffin wax. The thin sections of 5 μm were cut using a Rotary Microtome, mounted on glass slides, and stained for further histopathological examinations [21]. The histopathological images are obtained by EVOS XL Core Imaging System (Invitrogen -Thermo Fisher Scientific), Catalog number: AMEX1000; is a digital, transmitted light, inverted imaging system for cell and tissue culture applications. The high-quality optics, a 12.1′ high-resolution LCD display, and a digital colour camera deliver high-definition images for easy identification.

### Statistical analysis

Graph Pad Prism 5 software (Graph Pad Software Inc.) was used to calculate the standard deviation, two-way analysis of variance (ANOVA). The Pearson correlation coefficient and *p* values < 0.05 were regarded as significant. Values expressed are means of six replicate determinations standard deviation.

## Results

### LCMS

The LCMS chromatogram of plant extract profiling and metabolite identification of PRME showed the presence of various phytochemical derivatives with numerous characteristic peaks. With the help of the online LCMS database, nearly 80 peaks were identified, out of which 50 known molecules were evaluated with the help of chemical formula, mass, and m/z value. Almost 14 molecules show significant activity when compared with the reported literature (Fig. 1; Table 1).

**Table 1:** LCMS analysis and database search results of *P. rubiginosum* methanolic bark extract

Peak Number	Compounds	Formula	RT	Mass	m/z	DB diff (ppm)
1	3-Aminocaproic acid	C_6_H_13_NO_2_	1.256	131.0946	132.1019	− 0.1
2	3-O-Ethyl-L-ascorbic acid	C_8_H_12_O_6_	2.889	204.0615	209.0409	9.41
3	3,5,6-Trihydroxy-5-(hydroxymethyl)-2-methoxy-2-cyclohexen-1-one	C_8_H_12_O_6_	3.229	204.0615	209.041	9.37
4	Azocyclotin	C_20_H_35_N_3_Sn	3.291	429.1867	452.1755	2.93
5	Ethyl Oxalacetate	C_8_H_12_O_5_	3.523	188.0681	171.0649	1.72
6	13-Deoxycarminomycin	C_26_H_29_NO_9_	3.565	499.1891	482.1859	− 9.79
7	Validamycin B	C_20_H_35_NO_14_	3.798	513.2033	496.2003	4.76
8	Diphenyl disulfide	C_12_H_10_S_2_	4.238	218.0234	241.0128	− 4.78
9	Mahaleboside	C_15_H_16_O_8_	4.682	324.0838	307.0804	2.08
10	DL-Sulforaphane	C_6_H_11_NOS_2_	4.784	177.0244	182.0029	21.51
11	Octylamine	C_8_ H_19_ N	4.808	129.1519	130.1592	− 1.38
12	2-(4-Methyl-5-thiazolyl)ethyl formate	C_7_H_9_NO_2_S	5.106	171.0349	172.0422	2.73
13	Taraxinic acid glucosyl ester	C_21_H_28_O_9_	5.202	424.1726	407.1695	1.69
14	Cetraxate benzyl ester	C^24^ H_29_ NO_4_	5.311	395.2144	378.2125	0.77
15	3-Dimethylallyl-4-hydroxymandelic acid	C_13_H_16_O_4_	5.376	236.1036	219.1009	5.15
16	Isatidine	C_18_H_25_NO_7_	5.386	367.1641	390.1537	− 2.83
17	alpha-Hydrojuglone 4-O-b-D-glucoside	C_16_H_18_O_8_	5.742	338.0996	321.0962	1.6
18	Netilmicin	C_21_H_41_N_5_O_7_	5.782	475.2987	476.3056	4.04
19	2-[[(3a,5b,12a)-12-hydroxy-24-oxo-3-(sulfooxy)cholan-24-yl]amino]-Ethanesulfonic acid	C_26_H_45_NO_9_S_2_	6.207	579.2532	584.2313	0.62
20	Epicatechin 3-O-(4-methylgallate)	C_23_H_20_O_10_	6.853	456.1041	439.1006	3.48
21	Thiodiacetic acid sulfoxide	C_4_H_6_O_5_S	7.601	165.9906	170.9693	17.95
22	2-Naphthalenethiol	C_10_H_8_S	7.644	160.0341	183.0234	3.54
23	L-Pyridosine	C_12_H_18_N_2_O_4_	8.842	254.1279	259.1065	− 4.72
24	(2E)-Piperamide-C5:1	C_16_H_19_NO_3_	9.567	273.1359	256.1325	2.27
25	N1-Caffeoyl-N10-feruloylspermidine	C_26_H_33_N_3_O_6_	10.07	483.2305	466.2273	13.36
26	Rosmarinate	C_18_H_16_O_8_	10.234	360.0838	361.0911	2.11
27	23-Acetoxysoladulcidine	C_29_H_47_NO_4_	10.747	473.3581	478.3365	− 16.02
28	Butylparaben	C_11_H_14_O_3_	10.897	194.0937	195.101	3.07
29	L-Tryptophanamide	C_11_H_13_N_3_O	11.07	203.107	186.1034	− 5.41
30	Sulfadimidine	C_12_H_14_N_4_O_2_S	11.326	278.0857	279.0927	− 7.09
31	( +)-Prosopinine	C_18_H_35_NO_3_	11.33	313.2612	314.2681	1.49
32	E-64	C_15_H_27_N_5_O_5_	11.617	357.2018	362.1804	− 1.5
33	Mitragynine	C_23_H_30_N_2_O_4_	11.647	398.2216	403.2002	− 2.58
34	Phytosphingosine	C_18_H_39_NO_3_	12.02	317.2919	318.2991	3.3
35	Mitoxantrone	C_22_H_28_N_4_O_6_	13.37	444.2034	445.2106	− 5.69
36	Erinacine P	C_27_H_40_O_8_	14.117	492.273	493.2797	− 1.45
37	Glycine, N-[(3a,5b,7a)-3-hydroxy-24-oxo-7-(sulfooxy)cholan-24-yl]-	C_26_H_43_NO_8_S	14.245	529.2746	534.253	− 6.84
38	23-Acetoxysoladulcidine	C_29_H_47_NO_4_	15.004	473.3493	496.3383	2.64
39	Adenylosuccinic acid	C_14_H_18_N_5_O_11_P	15.008	463.0779	486.0668	− 8.25
40	Kanamycin C	C_18_H_36_N_4_O_11_	15.227	484.2371	507.2262	1.97
41	1-Methyl-6-(1,2,3,4-tetrahydro-6-hydroxy-2-naphthyl)-2(1H)-pyridone	C_16_H_17_NO_2_	15.243	255.1251	238.1217	3.4
42	Ganglioside GM3 (d18:1/22:1(13Z))	C_63_H_114_N_2_O_21_	15.325	1234.8142	631.391	− 18.42
43	Misoprostol (free acid)	C_21_H_36_O_5_	15.412	368.2555	351.2519	2.02
44	Chlorfenvinphos	C_12_H_14_Cl_3_O_4_P	15.762	357.9718	358.9789	− 6.37
45	Stigmast-22-ene-3,6-dione	C_29_H_46_O_2_	16.102	426.3497	409.3465	0.16
46	17-Hydroxylinolenic acid	C_18_H_30_O_3_	16.357	294.2189	277.2156	2.03
47	17-Hydroxylinolenic acid	C_18_H_30_O_3_	16.7	294.2189	277.2156	1.95
48	Chlozolinate	C_13_H_11_C_l2_NO_5_	16.933	330.9947	353.9846	20.21
49	Tetrahexosylceramide (d18:1/26:1(17Z))	C_70_H_128_N_2_O_23_	17.28	1364.8937	705.4359	− 2.16
50	Tetrahexosylceramide (d18:1/24:0)	C_68_H_126_N_2_O_23_	17.286	1338.8781	683.4228	− 2.21

m/z: mass-to-charge ratio; RT: retention time; DB diff (ppm): data base difference tool in parts per million

### Molecular docking

**Fig. 2:**
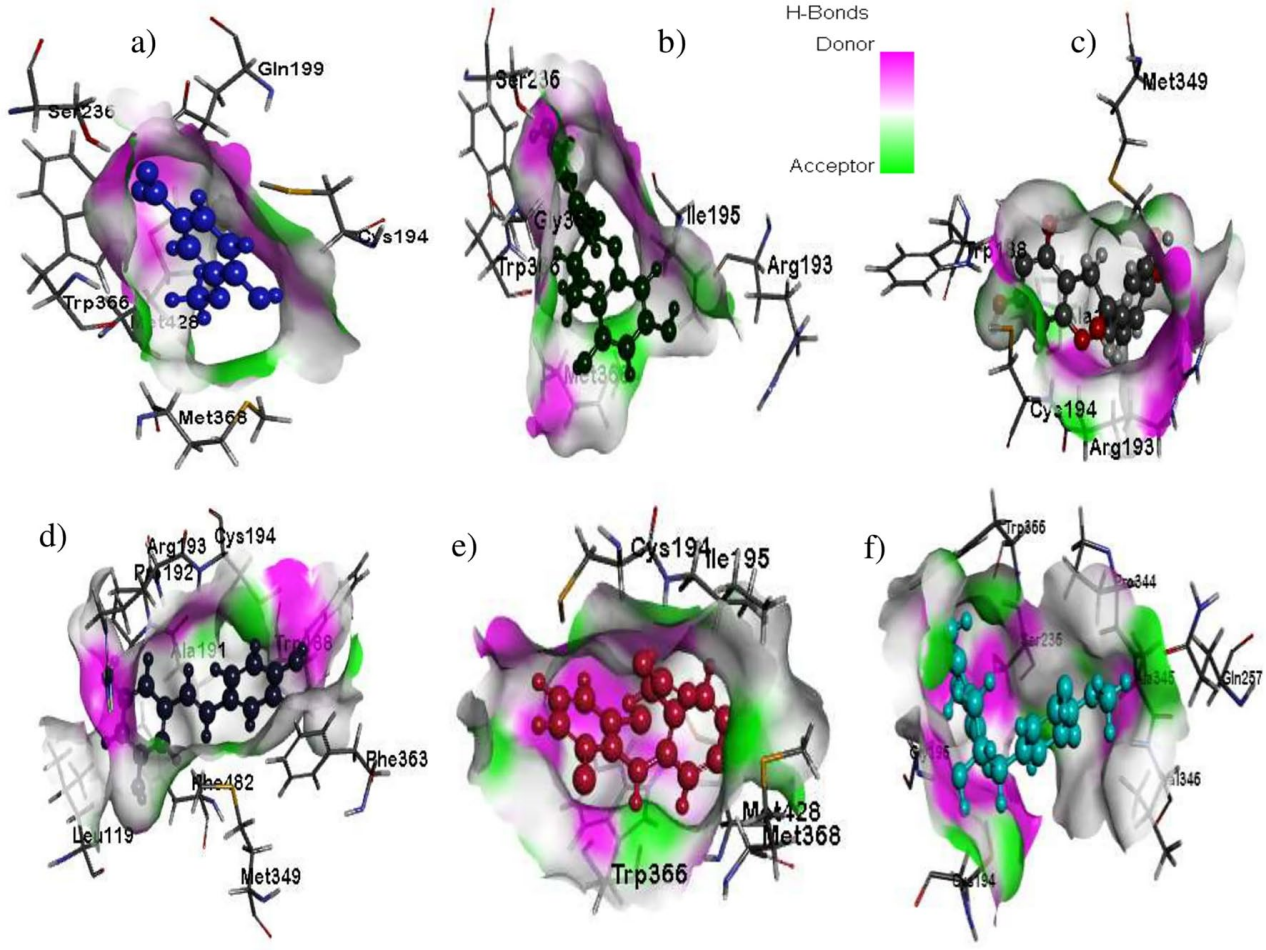
Inflammatory enzyme (iNOS) interaction with the ligand molecules isolated from PRME; Surface view interactions of iNOS protein with; **a** vanillic acid; **b** Gallocatechin; **c** Catechin; **d** *E*-reveratrol; **e** diclofenac sodium; **f** 4′-*O*-methylgallocatechin

**Fig. 3:**
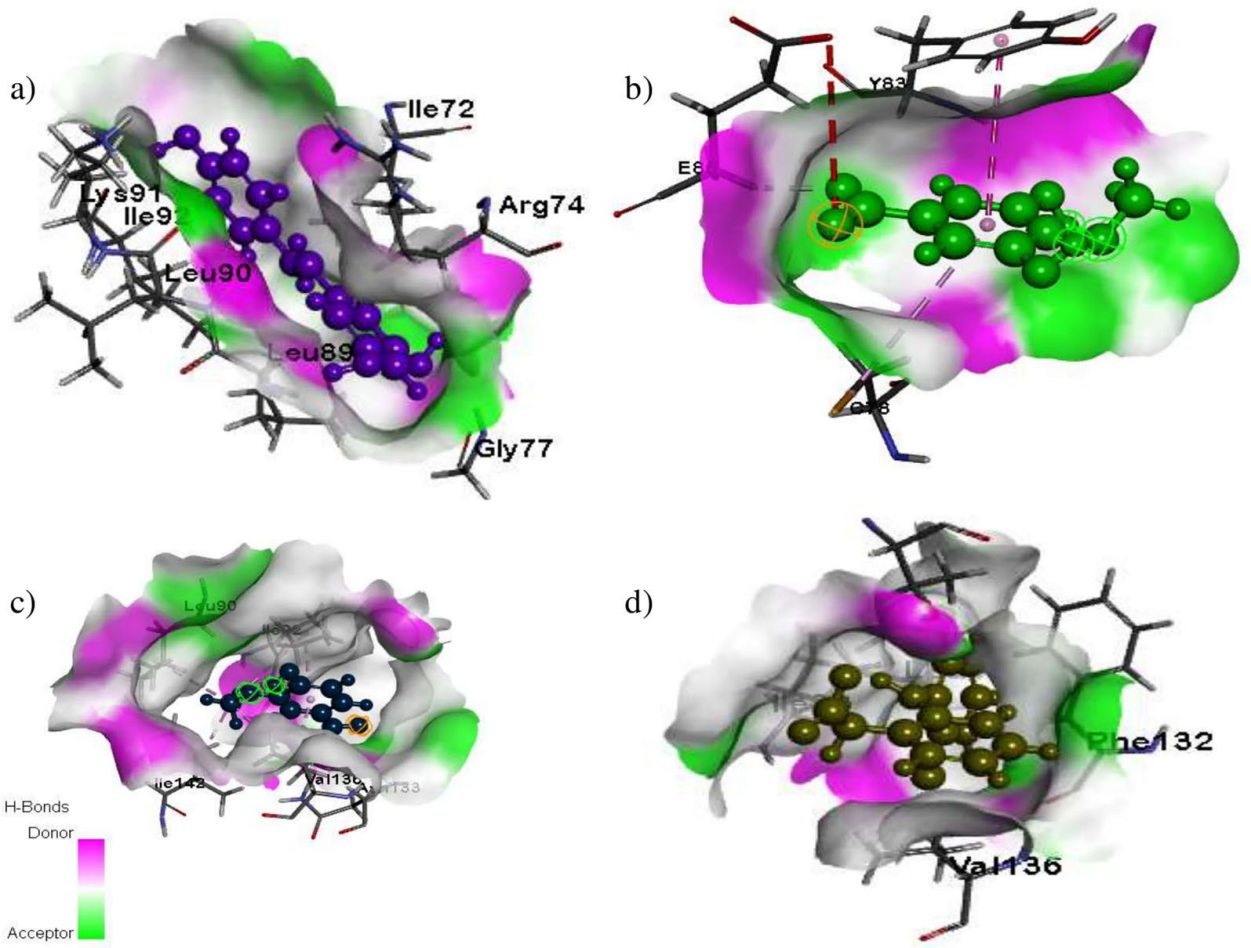
Pro-inflammatory cytokine (IL-6) interaction with the ligand molecules isolated from PRME; Surface view interactions of IL-6 protein with; **a** *E*-reveratrol; **b** 4-*O*-Methylgallic Acid; **c** Vanillic acid and **d** diclofenac sodium

Docking studies are commonly used to predict the molecular interactions, binding sites of ligands to protein molecules and to understand the 3D orientation pattern of stable protein–ligand combinations. This is commonly exploited in pharmacology to determine the binding pockets of drugs during their interaction with target sites, especially proteins. The Libdock score and binding energy of ligands against inducible nitric oxide synthases (iNOS) enzyme (Fig. 2) and Interleukins-6 (IL-6) cytokine (Fig. 3) were summarized in (Additional file 1: S.Tables 1, 2 Supplementary file attached).

### mRNA expression of inflammatory markers

**Fig. 4:**
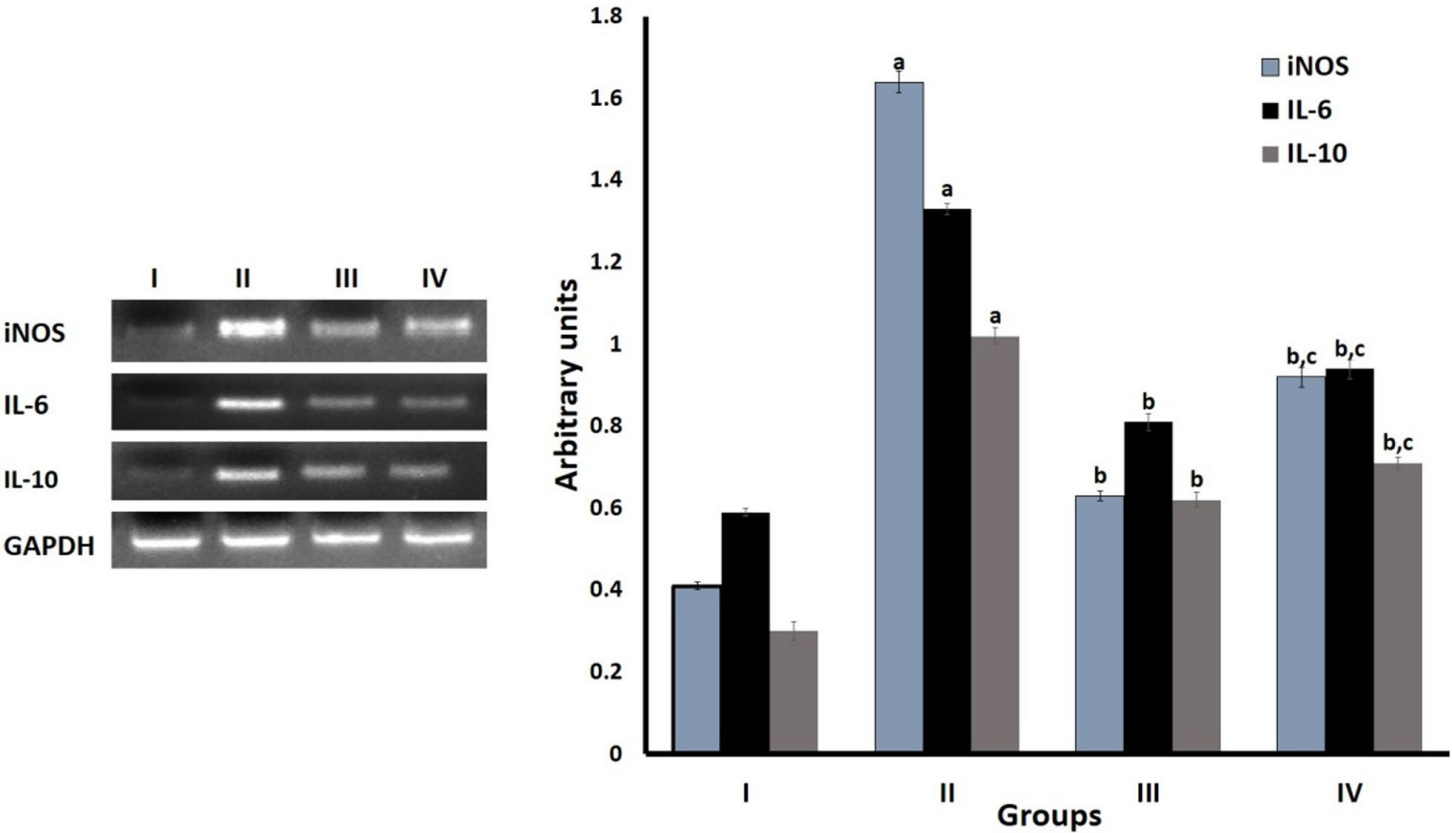
Graphical and photographic representations of mRNA expressions of iNOS, IL-6 and IL-10 on RAW 264.7 macrophages treated with PRME. Results were presented as mean ± SD, n = 4 with *p* ≤ 0.05. Group: I—control, group: II—LPS stimulated, group: III—LPS + PRME Group: IV—LPS + diclofenac sodium. I has been compared with II (‘a’ indicates values were significantly different from I), II is compared with III and IV (‘b’ indicates values were significantly different from II) and III is compared with IV (‘c’ indicates values were significantly different from III)

The relative expression of both the iNOS enzyme and anti-inflammatory cytokines IL-6 and IL-10 were upregulated in LPS treated group wheras PRME treated groups tolerated inflammation in comparison with standard drug (Fig. 4).

### Haematological parameters

The haematological parameters are commonly used to study the extent of the toxic effects of drugs, formulations, or plant extracts in laboratory animals. The toxic components are easily mixed with blood, being the circulating connective tissue, and can be identified by regular monitoring of these parameters. The different haematological parameters of PRME treated animals and control rats are evaluated and tabulated in (Table 2).

**Table 2:** Hematological parameters of PRME treated animals in acute toxicity study

Parameters	Group I	Group II	Group III	Group IV	Group V
RBC (10^6^/μL)	8.95 ± 0.34	9.01 ± 0.32	9.10 ± 0.34	9.11 ± 0.37	9.05 ± 0.28
WBC (10^3^/μL)	9.86 ± 1.24	10.00 ± 1.14	10.07 ± 1.21	11.14 ± 0.62	10.90 ± 0.86
Hb (%)	10.96 ± 1.53	11.11 ± 1.70	11.33 ± 1.69	11.85 ± 2.21	12.49 ± 1.53
PCV (%)	43.00 ± 2.37	42.86 ± 2.10	42.47 ± 2.72	41.36 ± 3.062	44.65 ± 2.08
PLT (10^3^/L)	896.39 ± 17.47	900.58 ± 17.85	903.16 ± 14.06	901.57 ± 13.58	907.78 ± 15.82

Values are expressed as a mean ± SEM (n = 6, and *P* < 0.05) RBC: red blood cell count; WBC: white blood cell count; Hb: haemoglobin concentration; PCV: Packed cell volume; PLT: platelet count

### Toxicity marker analysis

Renal functional markers such as serum urea, creatinine, and uric acid levels were assayed in PRME-treated animals and compared with the control group. No significant differences or any renal adverse effects were observed in treated animals. The synthesis capacity of hepatocytes was evaluated by accessing total protein levels and serum albumin. Both the markers are in a normal range, and no abnormality was observed (Table 3). Metabolic marker evaluation of serum total cholesterol and triglycerides did not show any notable variation in treated groups compared to a standard control, which indicated that PRME had no adverse effect on cholesterol metabolism. The antioxidant enzymes catalase and SOD are slightly increased in the PRME treated group compared to the normal group, which may be due to the potent antioxidants in PRME. The other stress parameters, such as glutathione peroxidise (GPx), glutathione content (GSH), and glutathione reductase (GRd), also showed a moderate increase in the PRME treated groups when compared to the normal saline-treated groups. Lipid peroxidation products [malonaldehyde (MDA), conjugated dienes (CD), and hydroperoxides (HP)] were found to be in a linear range in PRME-treated groups when compared to standard group (Table 4).

**Table 3:** Renal, liver, and metabolic marker evaluation in acute toxicity study

Parameters	Group I	Group II	Group III	Group IV	Group V
*Renal markers in serum*					
Urea (U/L)	40.49 ± 2.79	41.44 ± 2.89	42.78 ± 4.29	42.28 ± 3.06	44.71 ± 2.66
Creatinine (mg/dL)	0.56 ± 0.07	0.55 ± 0.08	0.58 ± 0.04	0.58 ± 0.05	0.61 ± 0.04
Uric acid (mg/dL)	0.96 ± 0.35	0.93 ± 0.47	1.10 ± 0.45	1.01 ± 0.38	1.16 ± 0.40
*Liver marker in serum*					
SGOT (U/L)	69.79 ± 4.48	69.86 ± 6.10	71.11 ± 5.88	73.47 ± 5.78	77.47 ± 6.90
SGPT (U/L)	20.15 ± 3.56	22.10 ± 4.40	23.51 ± 4.84	24.03 ± 4.04	26.72 ± 3.30
TP (g/dL)	5.93 ± 0.41	5.99 ± 0.30	5.96 ± 0.45	6.20 ± 0.42	6.12 ± 0.57
Albumin (g/dL)	3.03 ± 0.61	3.33 ± 0.56	3.40 ± 0.52	3.47 ± 0.53	3.34 ± 0.58
*Metabolic serum markers*					
Triglycerides (mg/dl)	42.60 ± 5.44	45.30 ± 6.05	48.57 ± 7.67	50.18 ± 6.38	51.82 ± 4.97
TC (mg/dl)	52.52 ± 5.10	59.58 ± 5.18	62.09 ± 7.50	63.16 ± 4.94	68.18 ± 4.90

Values are expressed as mean ± standard deviation (n = 6, and *P* < 0.05); Units per litre: U/L; milligram /decilitre: mg/dL; gram/deciliter: g/dL; serum glutamic-oxaloacetic transaminase: SGOT; Serum Glutamic Pyruvic Transaminase: SGPT; Total protein: TP; Total cholesterol: TC

**Table 4:** Antioxidant marker and lipid peroxidation product evaluation in acute toxicity study

Parameters	Group I	Group II	Group III	Group IV	Group V
*Antioxidant marker study in liver cells*					
GPx (mM/dl)	31.90 ± 2.02	33.00 ± 2.72	34.38 ± 2.12	34.80 ± 2.02	33.60 ± 2.09
GSH (mM/dl)	81.07 ± 3.03	83.00 ± 2.99	83.15 ± 3.44	84.02 ± 3.02	83.45 ± 2.52
GRd (mM/dl)	144.17 ± 5.20	143.11 ± 4.04	147.04 ± 5.21	145.40 ± 4.75	143.22 ± 4.12
SOD (U/dl)	2.45 ± 0.26	2.61 ± 0.31	2.55 ± 0.21	2.59 ± 0.54	2.90 ± 0.44
Catalase (mM/min/mg)	7.12 ± 0.45	7.57 ± 0.50	7.55 ± 0.46	7.47 ± 0.53	7.70 ± 0.43
*Lipid peroxidation products in liver cells*					
MDA (mM/dl)	2.56 ± 0.36	2.54 ± 0.26	2.58 ± 0.32	2.60 ± 0.36	2.71 ± 0.31
CD (mM/dl)	5.65 ± 0.39	5.54 ± 0.29	5.60 ± 0.44	5.54 ± 0.31	5.51 ± 0.34
HP(mM/dl)	7.95 ± 0.44	7.57 ± 0.27	7.64 ± 0.40	7.73 ± 0.43	7.90 ± 0.49

Values are expressed as mean ± standard deviation (n = 6). millimoles per decilitre: mM/dl; Units per decilitre: U/dl; millimolar per mins per milligram: mM/min/mg; millimoles per decilitre: mM/dl; Glutathione peroxidise: *GPx*, Reduced glutathione: GSH; Glutathione reductase: GRd; Sodium dismutase: SOD; Malonaldehyde: MDA; Conjugated dienes: CD; Hydroperoxides: HP

### Histopathological examination

**Fig. 5:**
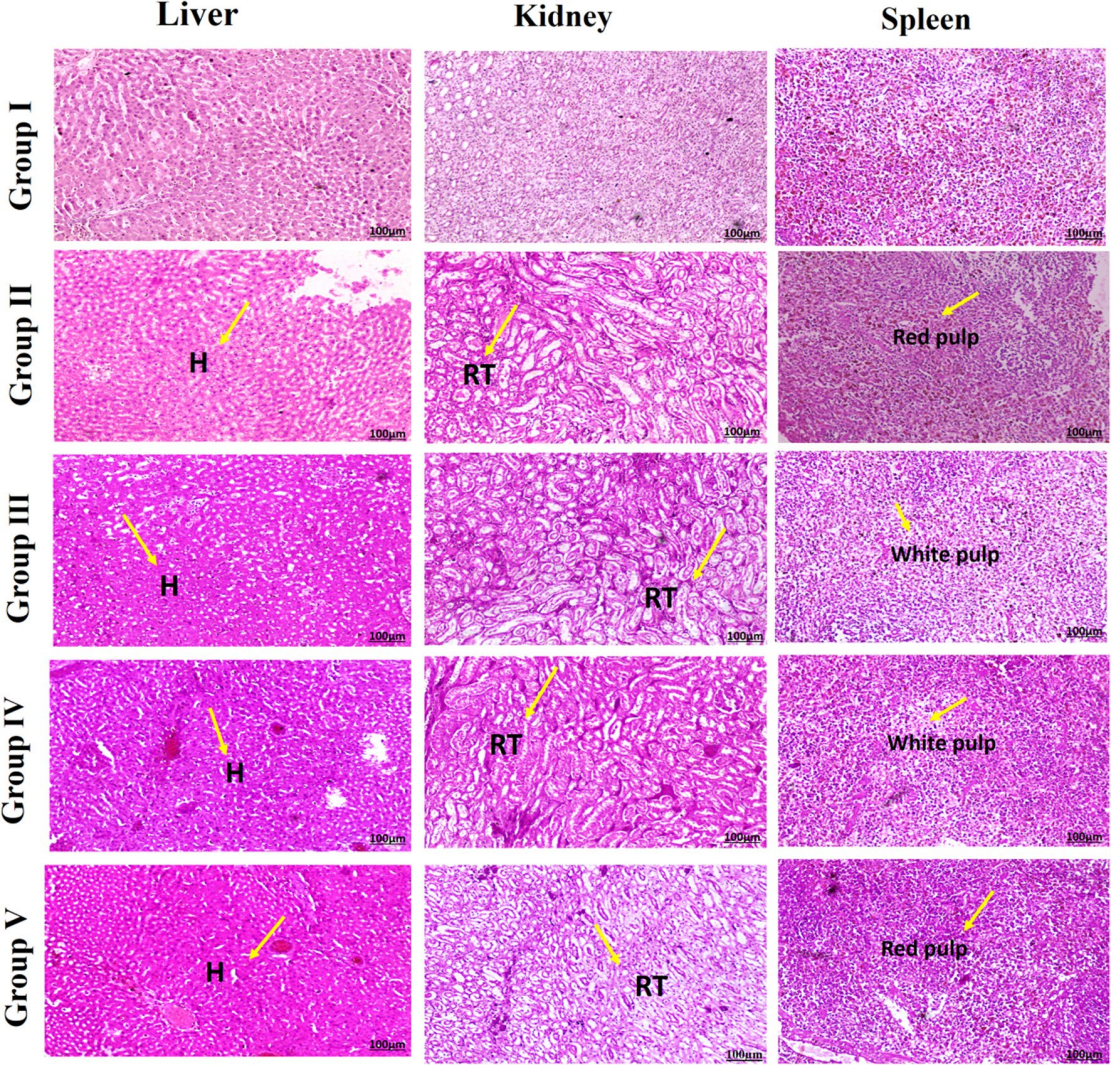
Histopathology sections of liver, kidney and spleen tissues during toxicity study; where; Group I—saline treated (normal); Group II—PRME 50 mg/kg/day; Group III—PRME 100 mg/kg/day; Group IV—PRME 500 mg/kg/day; Group V—PRME 1000 mg/kg/day treated groups

Histopathological examination is commonly used to understand the cellular level toxicity of plant-derived compounds or drugs during consumption. The detailed examination of hepatic and renal tissues in treated animals showed no notable changes in histology, cellular morphology, and overall tissue texture or cellular pattern when compared to normal saline administered rat tissues. This showed that PRME is non-toxic and would be a good candidate for long-term toxicity studies (Fig. 5).

## Discussion

In the Western Ghats, India, the traditional healers used diverse formulations and decoctions for treating bone fractures and associated inflammatory episodes. The tribal practitioners prepared the formulations for bone fracture treatment using a local alcoholic combination. To mimic the traditional alcoholic preparation, the methanolic fraction of bark extract was used for the study. In this study, *P. rubiginosum* was selected owing to its traditional use as an anti-inflammatory agent in formulations. Structural characterisation of *P. rubiginosum* bark extract was performed by NMR spectroscopy and revealed the presence of Vanillic acid, 4-*O*-Methylgallic acid, *E*-resveratrol, Gallocatechin, 4′-*O*-methylgallocatechin, and catechin [22].

LCMS is an analytical technique that combines high-performance liquid chromatography (HPLC), a powerful analytical separation technique, with mass spectroscopy, used in natural product chemistry and pharmaceutical research industries. LCMS is used for separating the analytes of interest based on combining the physical separation of molecules by liquid chromatography with the mass analysis capabilities of mass spectrometry. In the present analysis, we identified various phytochemicals in PRME, nearly 50 compounds with different molecular weights (m/z). Of these, 14 compounds seem to have significant biological activity based on published literature; compound 1: 3-aminocaproic acid, an antifibrinolytic agent used to induce clotting in post-operative conditions [23]. Compound 2: 3-O-Ethyl-L-ascorbic acid, used as an antioxidant and anti-ageing agent. Compounds 3,7,27, such as 3,5,6-Trihydroxy-5-(hydroxymethyl)-2-methoxy-2-cyclohexen-1-one, Validamycin B, and Rosmarinate, have antibacterial and antifungal properties [24, 25]. Compound 6: 13-Deoxycarminomycin, an anthracycline chemotherapeutic agent and Compound 35: Mitoxantrone, act as anti-cancerous agents [26, 27]. Compound 9: Mahaleboside is commonly used in the Unani system of medicine for treating rheumatoid arthritis [28]. Compound 10: DL-Sulforaphane derivatives are antioxidants and anti-inflammatory agents to prevent ageing and neurodegeneration [29]. Other sesquiterpene lactones, phenolic compounds, alkaloids, diterpenoids, indole-based alkaloids, steroidal alkaloids, and analgesic agents were isolated from PRME [30, 31]. This diverse variety of chemical constituents may be the reason behind the biological potency of PRME.

Molecular docking studies with murine inducible nitric oxide synthase oxygenase (1DD7) revealed an excellent binding affinity of Gallocatechin ligand -150.437 (Kcal/mol) due to the protein–ligand interactions of iNOS (A-chain) SER236, MET 368, ARG193, TRP366, and GLY365 amino acid residues and O-5, O-6, O-7 moiety of gallocatechin through hydrogen bonding. Similarly, the other ligands, such as catechin and 4′-O- methylgallocatechin, exhibited excellent hydrogen and hydrophobic interactions and are tabulated in (Additional file 1: Sup.Table 1). Vanillic acid showed a hydrogen bond of interactions with SER93 and ASN133 aminoacid residues of iNOS with O4 ligand moiety, with an excellent binding energy of − 38.2536 (Kcal/mol). Similarly, docking evaluation studies with the solution structure of mouse IL-6 (2L3Y) exhibited some remarkable binding affinity with *E*-resveratrol, vanillic acid and 4-O-methyl gallic acid compared to a standard molecule. The libdock score values were found to be in the order of *E*-resveratrol > vanillic acid > diclofenac sodium > 4-*O*-methylgallic acid (Additional file 1: Sup.Table 2). The docking analysis showed that the gallocatechin and E-resveratrol present in PRME exhibited excellent inhibition against the inflammatory enzymes iNOS and IL-6.

The MTT assay showed good cellular viability of PRME up to a concentration of 100 µg/ml, and the LC_50_ value was found to be 106.869 µg/ml in RAW 264.7 cells [32]. LPS activates iNOS and releases nitric oxide (NO) by activating nuclear factors, including NF-kB. In LPS treatment on RAW 264.7 cells, LPS stimulated the macrophages and increased the release of iNOS enzymes and expression up to a range of 1.6 arbitrary units, a fourfold increase compared to the control group. Along with the iNOS, LPS induces the production of pro-inflammatory cytokines, such as interleukin (IL)-6 and anti-inflammatory cytokines, such as IL-10. As a crucial player in inflammation, NO is an intermediate molecule in immune responses including autoimmune processes and chronic degenerative diseases. The cytokine level is enhanced in chronic inflammatory disease [33]. PRME administration can tolerate these inflammatory markers and significantly downregulated gene expression by nearly 0.6 arbitrary units compared to the standard (diclofenac sodium) treated group (Fig. 4). The gene expression studies support the anti-inflammatory ability of PRME same as revealed by molecular docking results.

The current study analysed the safety level and effective optimal dosage of PRME in experimental healthy female SD rats. Cage-side observations are a good measure of an animal’s overall health. Any change in behaviour, food-water intake, stomach distension, urine colour, and consistency are critical indicators of toxicity in test animals. No apparent signs of toxicity were seen during the investigation. All the animals were healthy and had an excellent gait after PRME treatment. The physiological health of the animal plays a crucial role in diet intake [34]. There was a linear increase in total weight gain between dose ranges from 50 to 1000 mg/body weight, showing an initial weight of 221.16 ± 7.12 g and an end weight of 260.33 ± 5.01 g. After 14 days of PRME treatment, the final weight ranged from 223.33 ± 6.43 to 269.5 ± 0 5.96 g, respectively. The treated animals were sacrificed on the 15th day of the experiment in order to obtain blood for biochemical studies. There is no remarkable variation between healthy and PRME-treated rats in red blood cell count, white blood cell count, haemoglobin concentration, packed cell volume, and platelet count, demonstrating that the PRME extracts are compatible with treated animals in various blood parameters up to 1000 mg/kg body weight (Table 2).

Liver enzymes are proteins that initiate various chemical reactions in our body. SGPT and SGOT are the critical enzymes enhanced during cellular damage, especially during liver tissue degeneration and necrosis [35], and both enzymes were found to be in the optimal range in the PRME-treated groups. PRME-treated rats showed no notable variation in liver markers, indicating they could be used in long-term toxicity investigations (Table 3). A renal function test was used to determine the kidney's functional capacity by determining urea, creatinine, and uric acid. The detection of serum urea is one of the most reliable clinical indicators for determining the kidney's health. With PRME treatment, the urea ranged from (41.44 ± 2.89 to 44.71 ± 2.66 U/L) and showed closed linearity with the control animals (40.49 ± 2.79 U/L). Compared with the control group animal values (0.96 ± 0.35 mg/dL), no significant changes in serum uric acid values were observed in the PRME treatment groups (0.93 ± 0.47 to 1.16 ± 0.40 mg/dL). The serum creatinine measurement is an approximate indicator of glomerular filtration rate (GFR) and kidney function [36]. The creatinine levels in the PRME-administered groups ranged from (0.55 ± 0.08 to 0.61 ± 0.04 mg/dL) to the normal rats' value (0.56 ± 0.07 mg/dL). The RFT findings suggest that PRME is non-toxic, and the bark extract is a good candidate for detailed biological study.

Serum cholesterol and triglycerides measurements showed no significant variation in PRME-treated groups, up to a concentration of 1000 mg/kg body weight, and can be considered safe (Table 3). Studies on stress markers are commonly used to examine how internal and external factors affect an organism's normal homeostasis at the molecular level [37]. Hepato-renal tissues are protected against oxidative damage by superoxide dismutase, a key enzyme that eliminates reactive oxygen species from the body. There was a remarkable increase in superoxide dismutase (SOD) activity in the PRME-administered group, which showed a positive antioxidant mechanism against stress in the body. Catalase (a degradation enzyme) is typically involved in eliminating excess H_2_O_2_ and may contribute to the anti-oxidative activity of PRME, which is due to its high phenolic content [11]. The enzymatic antioxidants, including SOD, catalase (CAT), glutathione peroxidase (GPX), glutathione reductase (GR/GRD), and glutathione (GSH), were found to be in optimum in all the treated groups, thus confirmed the therapeutic potential of PRME in in vivo conditions.

The level of lipid peroxidation is commonly determined as a significant indicator of oxidative stress. MDA is formed when unsaturated fatty acids in phospholipids are oxidised, causing damage to cell membranes [38]. From Table 4, the MDA values in the PRME-administered groups do not show significant variations. Similarly, hydroperoxide (HP) and conjugated diene (CD) values are within the normal range compared to the PRME-treated group. Based on the lipid peroxidation product evaluation, we concluded that PRME does not cause any oxidative stress to the treated animals. Selenium-dependent GPx and selenoprotein P (SeP) optimise the decomposition of hydroperoxides [39]. The GPx values of the PRME group and control are very similar to the GSH content and act as reductants for converting H_2_O_2_ or organic hydroperoxides to water or the corresponding alcohols. These findings show that PRME can alleviate oxidative stress and be employed in more extensive studies. Different internal and external features impact the well-maintained homeostasis of living creatures at the molecular level, resulting in cellular stress [39]. During the histopathological examination, the PRME-treated animal liver, spleen, and renal tissue were found to be of normal texture and no significant alterations were observed.

## Conclusions

The LCMS analysis revealed the presence of a diverse variety of phytocompounds in PRME, and the excellent biological efficacy of this medicinal bark extract is found to be due to its phytochemical content. On LPS treatment, the macrophages activate and release cytokines and other inflammatory mediators to promote the inflammatory process and undergo apoptosis. PRME administrations can significantly downregulate inflammatory markers, especially inflammatory cytokines. The computational analysis of iNOS and IL-6 also supports the gene expression results. In the dose-dependent study conducted on healthy SD rats, PRME was found to be non-toxic up to a concentration of 1000 mg/kg body weight for 14 days. The lowest non-toxic dose of 50 mg/kg body weight was more effective and selected for further detailed biological study.

### Supplementary Information

**Additional file 1**. Molecular docking interactive studies with inflammatory markers and ligand molecules isolated from PRME.

## Data Availability

All experiment data during this study are included in this manuscript.

## Abbreviations

CD: Conjugated diene
FAO: Food and agriculture organization of the United Nations
GSH: Glutathione content
GPx: Glutathione peroxidises
HP: Hydroperoxide
LCMS: Liquid chromatography mass spectrometry
iNOS: Inducible nitric oxide synthase
IL-6: Interleukin-6
IL-10: Interleukin-10
LFT: Liver function tests
MDA: Malonaldehyde
PRME: *Pterospermum rubiginosum* methanolic bark extract
PCV: Packed cell volume
RFT: Renal function tests
RBC count: Red blood cell count
SD rats: Sprague Dawley rats
SeP: Selenoprotein P
SGOT: Serum glutamic oxaloacetic transaminase
SGPT: Serum glutamic pyruvic transaminase
SOD: Sodium dismutase
WBC count: White blood cell count
WHO: World Health Organization
