# Supplementary material for: In silico, anti-inflammatory and acute toxicological evaluation of an indigenous medicinal plant Pterospermum rubiginosum using Sprague-Dawley rats
Source: Lab Anim Res. 2024 Feb 7;40:2. doi: 10.1186/s42826-024-00191-w (PMC10848399; doi:10.1186/s42826-024-00191-w)
Supplement: Supplementary file 1 — Additional file 1. Molecular docking interactive studies with inflammatory markers and ligand molecules isolated from PRME. [file 42826_2024_191_MOESM1_ESM.docx]

Supplementary file.

Tables

1. iNOS enzyme interactions with the ligand molecules isolated from PRME
2. IL-6 (interleukins) interactions with the ligand molecules isolated from PRME

| Nos | Compound | LibDock Score | Interacting Residues | Bond Distance | Nature of Bonding |
| --- | --- | --- | --- | --- | --- |
| 1 | Catechin | 98.6595 | A:ARG193:HN - 73160:O1  73160:H23 - A:MET349:SD  A:TRP188:HA - 73160:O4  A:CYS194:SG - 73160  A:MET349:SD - 73160  A:MET349:SD - 73160  A:ALA191 - 73160  A:ARG193 - 73160  A:CYS194 - 73160  73160 - A:ALA191  73160 - A:ALA191  73160 - A:ARG193 | 2.31079  2.74507  2.33884  3.1273  5.62084  5.84905  4.07437  5.02884  4.21315  3.28842  4.38013  4.76399 | Hydrogen Bond  Hydrogen Bond  Hydrogen Bond  Other  Other  Other  Hydrophobic  Hydrophobic  Hydrophobic  Hydrophobic  Hydrophobic  Hydrophobic |
| 2 | Gallocatechin | 107.086 | A:SER236:HG - 65084:O6  A:SER236:HG - 65084:O7  65084:H23 - A:MET368:SD  65084:H25 - A:ARG193:O  A:GLY365:HA1 - 65084:O5  A:GLY365:HA1 - 65084:O7  65084:H27 - A:TRP366  65084:H27 - A:TRP366  A:MET368:SD - 65084  A:ILE195 - 65084  A:MET368 - 65084  65084 - A:ILE195 | 2.55751  1.60872  2.93087  2.51937  2.3342  2.31255  2.82571  2.6037  3.47238  5.32942  4.64506  4.7767 | Hydrogen Bond  Hydrogen Bond  Hydrogen Bond  Hydrogen Bond  Hydrogen Bond  Hydrogen Bond  Hydrogen Bond  Hydrogen Bond  Other  Hydrophobic  Hydrophobic  Hydrophobic |
| 3 | 4'-*O*- methylgallo catechin | 93.3421 | A:CYS194:HG - 10087345:O1  A:GLY196:HN - 10087345:O2  A:SER236:HG - 10087345:O4  10087345:H25 - A:TRP366:O  A:ALA345:HA - 10087345:O5  10087345:H38 - 10087345:O6  10087345:H39 - A:GLN257:OE1  A:CYS194:SG - 10087345  A:TRP366 - 10087345  A:CYS194 - 10087345  10087345:C23 - A:PRO344  10087345:C23 - A:VAL346 | 1.97837  1.89008  2.40735  2.05501  2.96038  1.78694  2.54465  5.49819  4.67872  4.63015  4.32333  4.50245 | Hydrogen Bond  Hydrogen Bond  Hydrogen Bond  Hydrogen Bond  Hydrogen Bond  Hydrogen Bond  Hydrogen Bond  Other  Hydrophobic  Hydrophobic  Hydrophobic  Hydrophobic |
| 4 | *E*-Resveratrol | 83.9342 | A:PHE363 - 445154  A:PHE482 - 445154  445154 - A:LEU119  445154 - A:ALA191  445154 - A:PRO192  445154 - A:ARG193  445154 - A:ALA191  445154 - A:CYS194  445154 - A:MET349 | 4.14415  4.93915  5.25874  4.35015  5.42858  5.21549  5.38942  4.01671  5.16095 | Hydrophobic  Hydrophobic  Hydrophobic  Hydrophobic  Hydrophobic  Hydrophobic  Hydrophobic  Hydrophobic  Hydrophobic |
| 5 | Diclofenac sodium | 77.3247 | 5018304:H20 - A:TRP366:O  A:CYS194:HA - 5018304:O3  5018304:O3 - 5018304  A:TRP366 - 5018304  5018304 - A:ILE195  5018304 - A:MET368  5018304 - A:MET428  5018304 - A:CYS194 | 1.72174  2.15692  4.943  5.41907  5.22648  4.46071  4.73785  5.37281 | Hydrogen Bond  Hydrogen Bond  Electrostatic  Hydrophobic  Hydrophobic  Hydrophobic  Hydrophobic  Hydrophobic |
| 6 | Vanillic acid | 69.9857 | A:SER93:HG - 8468:O4  A:ASN133:HD21 - 8468:O4  8468:C12 - A:LEU90  8468:C12 - A:ILE92  8468:C12 - A:VAL136  8468:C12 - A:ILE142  8468 - A:ILE92  8468 - A:VAL136 | 2.3572  2.40176  4.46695  4.91004  4.69256  3.60651  3.67427  3.61149 | Hydrogen Bond  Hydrogen Bond  Hydrophobic  Hydrophobic  Hydrophobic  Hydrophobic  Hydrophobic  Hydrophobic |

**Table 1 iNOS enzyme interactions with the ligand molecules isolated from PRME**; where; Number-Nos

| Nos | Compound | LibDock Score | Interacting Residues | Bond Distance | Nature of Bonding |
| --- | --- | --- | --- | --- | --- |
| 1 | *E*-Resveratrol | 82.6942 | A:GLY77:HN - 445154:O2  445154:H20 - A:LYS91:O  A:LEU90:O - 445154  445154 - A:ARG74  445154 - A:LEU89  445154 - A:ILE72  445154 - A:ILE92 | 2.56951  2.36146  2.57656  3.40563  4.12228  4.09508  5.02067 | Hydrogen Bond  Hydrogen Bond  Other  Hydrophobic  Hydrophobic  Hydrophobic  Hydrophobic |
| 2 | Diclofenac sodium | 69.8837 | A:THR187:HB - 5018304:O3  A:VAL136:HB - 5018304  A:PHE132 - 5018304  5018304:Cl1 - A:ILE92  A:PHE132 - 5018304:Cl1  5018304 - A:ILE92  5018304 - A:LEU96 | 2.84828  2.74662  4.87539  4.12893  4.35259  3.85994  5.20544 | Hydrogen Bond  Hydrophobic  Hydrophobic  Hydrophobic  Hydrophobic  Hydrophobic  Hydrophobic |
| 3 | Vanillic acid | 70.9467 | A:SER93:HG - 8468:O4  A:ASN133:HD21 - 8468:O4  8468:C12 - A:LEU90  8468:C12 - A:ILE92  8468:C12 - A:VAL136  8468:C12 - A:ILE142  8468 - A:ILE92  8468 - A:VAL136 | 2.3572  2.40176  4.46695  4.91004  4.69256  3.60651  3.67427  3.61149 | Hydrogen Bond  Hydrogen Bond  Hydrophobic  Hydrophobic  Hydrophobic  Hydrophobic  Hydrophobic  Hydrophobic |
| 4 | 4-*O*-Methylgallic acid | 52.5794 | A:GLU86:HA - 78016:O5  A:TYR83 - 78016  78016 - A:CYS78 | 2.24222  5.09982  5.45012 | Hydrogen Bond  Hydrophobic  Hydrophobic |

**Table 2 IL-6 (interleukins) interactions with the ligand molecules isolated from PRME**; where; Nos: Number.
